# Bioaccessibility of Phenolic Compounds from Mistletoe Infusions and Effect of In Vitro Digestion on Its Antioxidant and Pancreatic Lipase Inhibitory Activity

**DOI:** 10.3390/foods11213319

**Published:** 2022-10-23

**Authors:** Erick Paul Gutiérrez-Grijalva, Victor Eduardo Zamudio-Sosa, Laura Aracely Contreras-Angulo, Nayely Leyva-López, J. Basilio Heredia

**Affiliations:** 1 Cátedras CONACYT-Centro de Investigación en Alimentación y Desarrollo, A.C. Carretera a Eldorado Km. 5.5, Col. Campo El Diez, Culiacán 80110, Sinaloa, Mexico; 2 Centro de Investigación en Alimentación y Desarrollo, A.C. Carretera a Eldorado Km. 5.5, Col. Campo El Diez, Culiacán 80110, Sinaloa, Mexico

**Keywords:** antioxidants, bioaccessibility, in vitro digestion, mistletoe, pancreatic lipase, phenolic acids, polyphenols

## Abstract

*Phoradendron brachystachyum* is an American mistletoe distributed in México and used ethnobotanically in infusions to treat hypertriglyceridemia and lower cholesterol levels. This study aimed to evaluate the bioaccessibility of the phenolic acids from mistletoe infusions and the effect of simulated digestion on its antioxidant and lipase inhibitory properties. The in vitro digestion process decreased the antioxidant capacity activity by the TEAC and ORAC assays in infusions from leaves, stems, and whole plant samples. Moreover, the individual phenolic content of mistletoe infusions was also affected by the in vitro digestion process; the most abundant individual phenolic constituents at the end of the digestion process were ferulic and quinic acids. These compounds showed low bioaccessibility values ranging from 7.48% to 22.60%. In addition, the in vitro digestion diminished the pancreatic lipase inhibition percentage of leaves and whole plant infusions but increased it in the stem samples. This research showed that given the phenolic content and pancreatic lipase inhibitory activity of mistletoe infusions, it could be used as a potential source for the development of functional foods and nutraceuticals; nonetheless, its phenolic content is affected by gastrointestinal digestion; thus, encapsulation strategies are encouraged to protect these metabolites from the gastrointestinal environment while preserving their antioxidant and hypolipidemic potentials.

## 1. Introduction

Metabolic syndrome (MetS) is a cluster of metabolic factors, such as abdominal obesity, insulin resistance, hypertension, and dyslipidemia, that increase the risk of cardiovascular diseases and diabetes. The World Health Organization states that cardiovascular diseases and diabetes are among the leading causes of death worldwide [1]. The treatment of MetS focuses on lifestyle changes and pharmacological drugs. Some of the most commonly prescribed drugs aim to inhibit enzymes involved in carbohydrate and lipid metabolism such as α-glucosidase and pancreatic lipase; these enzymes break down sugars and lipids to ease their absorption; thus, drugs such as acarbose and orlistat are often prescribed with this purpose [2]. Nonetheless, people with MetS often engage in long-term use of these drugs, which can cause adverse effects such as decreased vitamin E absorption, abdominal pain, and liver failure; thus, new active agents have been evaluated from different plant samples as a potential adjuvant, or to identify the active ingredients that can exert a therapeutic effect against metabolic syndrome [3,4].

Mistletoe is the name given to diverse groups of parasitic plant species mainly comprised of five botanical families Loranthaceae, Misodendraceae, Santalaceae, Amphorogynaceae, and Viscaceae. American and European mistletoe species have been used in folk medicine against cancer as hypoglycemic, hypolipidemic, and hypotensive agents [5]. Ethnopharmacological studies have shown that mistletoe has antioxidant, antiproliferative, anti-inflammatory, antidiabetic, antihypertensive, and antimicrobial properties [5,6]. These bioactivities have been associated with phytochemicals such as terpenes, alkaloids, and phenolic compounds [7,8].

Mistletoes are among the different plant matrices with lipase enzyme inhibitory potential [9,10]. The species *Phoradendron brachystachyum*, commonly known as “toji”, is a mistletoe belonging to the Santalaceae family. This species is widely distributed throughout Mexico in urban and wild areas [11]. The genus *Phoradendron* has been widely used ethnobotanically in the form of infusions. There are reports that it has been used to treat stomach pain and digestive disorders such as diarrhea, diabetes, hypertension, and cancer and as an immunomodulator [12].

A chemical analysis of an acetone extract of *P. brachystachyum* identified 20 different phytochemical compounds, including 12 triterpenes, two linear hydrocarbons, four sterols, and two flavonoids [11]. *Phoradendron reichenbachianum* is another mistletoe of the same genus, which has been analyzed for its content of phytochemical compounds. Its content highlights ursolic acid, moronic acid, morolic acid, and oleanolic acid, which are also identified in *P. brachystachyum*. Oleanolic acid, found in the genus *Phoradendron*, has been highlighted for its antihyperlipidemic and anti-atherosclerotic activity [13,14]. On the other hand, a study carried out in diabetic rats using moronic and morolic acids as treatment at doses of 50 mg/kg for 10 days showed a significant reduction in plasma values of total cholesterol and triglycerides, as the basal values of a group of normal rats [15].

Additionally, it is important to mention that phenolic compounds usually have low bioaccessibility as they undergo chemical transformations during their passage through the gastrointestinal environment. In this sense, the bioaccessibility of phenolic compounds can be evaluated by in vitro digestion methods to simulate the physiological and biochemical conditions in the mouth, stomach, and small intestine [16,17]. On this subject, *Morus alba* leaf extracts are rich in caffeoylquinic acid and quercetin; after in vitro digestion, these compounds show that their bioaccessibility is maintained at 68.39% and 30.07%, respectively [18]. On the other hand, the red chiltepin (*Capsicum annuum* L. var. *glabriusculum*), widely distributed throughout México, is rich in phytochemical compounds and, like *Morus alba*, shares the presence of quercetin. However, a methanol/water extract from the red chiltepin fruit showed that when subjected to in vitro digestion, its quercetin content decreased to 18.97% [19]. Here, the authors showed that the bioaccessibility of p-coumaric acid, quercetin, and luteolin is influenced by their interactions with the food matrix, changes in the extraction method, and the solvent used [19].

There are currently no specific studies on the bioaccessibility or bioavailability of phytochemical compounds from *P. brachystachyum* or any other mistletoe species. Thus, in this study, we aimed to evaluate the bioaccessibility of phenolic compounds from a regionally consumed mistletoe species, *P. brachystachyum,* in the form of infusions, as it is commonly consumed. Furthermore, we assessed the effect of an in vitro digestion process on its antioxidant and pancreatic lipase inhibition activity.

## 2. Materials and Methods

### 2.1. Plant Material

The mistletoe was collected in Guamúchil, Sinaloa (254,442, −1,080,522), parasitizing mesquite trees (*Prosopis juliflora*). The plants were identified at the herbarium Jesús González Ortega of the School of Agriculture from the Universidad Autónoma de Sinaloa. The identification catalog numbers for the mistletoe and mesquite tree were FA-UAS-022352 and FA-UAS-013284, respectively. The mistletoe samples were separated into leaf, and stem, also a set of the whole plant was used. They were then washed in chlorinated water at 50 ppm and allowed to dry at 40 °C for 48 h in an Excalibur Food Dehydrator Parallax Hyperware (Sacramento, CA, USA). The dried sample was recovered, and leaves, stems, and whole plant samples were ground separately in a Turf brand coffee mill until a fine powder was obtained. The ground powder was passed through a sieve of 600 μm. It was collected in airtight plastic bags and sealed in cellophane bags to prevent moisture from passing into the sample.

### 2.2. Preparation of Mistletoe Infusions

The powder of mistletoe samples was used to prepare infusions following the report of Chen et al. [20] with modifications. First, 50 mL of distilled water was added to 1 g of *P. brachystachyum* powder, and then samples were placed in an orbital shaker at 80 rpm for 2 h. After that, samples were placed in a water bath at 60 °C for 20 min. Next, the mixture was cooled down at room temperature and centrifuged at 3000 rpm for 10 min, and the supernatant was collected.

### 2.3. Simulated Gastrointestinal In Vitro Digestion

An in vitro gastrointestinal digestion process was performed following the indications of the INFOGEST method [17] with modifications. This process consists of a 3-step procedure that simulates the physiological and pH conditions in the mouth, stomach, and small intestine; the digestion is performed at 37 °C. At the end of the digestion process, the samples were centrifuged at 10,000 rpm for 15 min at 4 °C, and the supernatant was collected. After that, the supernatant was kept at −20 °C to stop the digestive reactions. Then, the samples were freeze-dried at −50 °C and a pressure of 0.070 mBar in a Labconco Freezone 18 (Labcon Co., Kansas, MO, USA). Finally, lyophilized samples were resuspended in distilled water.

### 2.4. Identification and Quantification of Phenolic Acids by UPLC-qToF-MS/MS

The phenolic acids were evaluated based on a previous report [21]. Briefly, the identification and quantification of phenolic acids were performed using a UPLC class H, coupled to G2-XS QTof mass analyzer (quadrupole and time of flight) (Waters Corporation, Santa Clara, CA, USA), through a capillary: 1.5 kV and sampling cone: 30, with solvation of 800 (L/h) at a temperature of 500 °C, and an electrospray ionization (ESI) source. Phenolic acids from the undigested and digested samples were separated using a UPLC BEH C18 column (1.7 µm × 2.1 µm × 100 mm (Waters Corporation) at 40 °C. Gradient elution was conducted with acidified water (A) (water-formic acid 0.1%) and acetonitrile (B) at a flow rate of 0.30 mL/min and 1 µL injection volume. The following gradient was used 0 min, 95% A; 5 min, 70% A; 9 min, 30% A; 10 min, 0% A; 11 min, 95% A; 11.5 min, 95% A. The ionization of the phenolic acids was carried out by electrospray in negative mode (ESI-), with a capillary voltage of 1.5 kV, sampling cone: 30 V, desolvation gas of 800 (L/h), and a temperature of 500 °C. A 0–30 V collision ramp was used. The Massbank of North America (MoNA) database was used to identify compounds. For the quantification, curves of phenolic acids (caffeic acid, chlorogenic acid, p-coumaric acid, gallic acid, quinic acid, and ferulic acid) were used. Measurements were made in triplicate (n = 3).

### 2.5. Bioaccessibility of Phenolic Acids from Mistletoe Infusions

The bioaccessibility percentage under simulated gastrointestinal digestion was determined using the following equation:

Bioaccessibility (%) = (Final concentration/Initial concentration) × 100

where the final concentration is the phenolic content at the end of the gastric and intestinal phases, and the initial concentration is the phenolic content in the undigested samples.

### 2.6. Antioxidant Capacity Assays

#### 2.6.1. Trolox Equivalent Antioxidant Capacity (TEAC)

The TEAC assay was performed following the methodology reported by Thaipong et al. [22] with modifications reported in Picos-Salas et al. [23]. First, the ABTS+ radical was formed by adding 2.4 mM potassium persulphate at a 1:1 ratio and incubating the mixture at room temperature in the absence of white light for 12–16 h before use. Next, the radical was diluted in ethanol to an absorbance of 0.7 at a wavelength of 734 nm. The assay began when 10 μL of the sample was mixed with 190 μL of ABTS+ and incubated for 2 h. After that, absorbance was measured at 734 nm using a microplate Synergy HT spectrophotometer (Synergy HT, Bio-Tek Instruments, Inc., Winooski, VT, USA). The ABTS+ radical was used as blank, and the results were calculated using a Trolox standard curve and expressed as mmol of Trolox equivalents per mL infusion (mmol TE/mL).

#### 2.6.2. Oxygen Radical Absorbance Capacity (ORAC) Assay

The ORAC assay was performed as mentioned by Huang et al. [24] with some modifications. The reaction mixture consisted of 25 μL of the sample, 50 μL of 95.8 μM 2,2′-azobis(2-methylpropionamidine) dihydrochloride (AAPH), and 150 μL of 0.96 μM fluorescein contained in a black-walled, clear-bottom 96-well microplate. Phosphate buffer was used as a blank, and AAPH was added to start the reaction. The loss of fluorescence was measured every 70 s for 70 min at 485 nm for excitation and 580 nm for emission using a Synergy HT spectrophotometer. Results were calculated using a regression equation describing the relationship between the Trolox concentration and the net area under the fluorescein decay curve. Results are expressed as μmol of Trolox equivalents (TE) per mL (μmol TE/mL).

### 2.7. Determination of Total Reducing Capacity and Flavonoid Content

The total reducing capacity of mistletoe samples was determined by the Folin–Ciocalteu method adapted from Swain and Hillis [25]. Briefly, 10 μL of the sample was mixed with 230 μL of distilled water and 10 μL of 2N Folin–Ciocalteu reagent in a 96-well microplate and left to incubate for 3 min. After that, 25 μL of Na_2_CO_3_ was added and incubated for 2 h in the absence of white light. Finally, absorbance was measured at 725 nm in a microplate reader EPOCH BioTek (Biotek Instruments, Inc., Winooski, VT, USA). Total reducing capacity was quantified using a gallic acid standard curve (0, 0.05, 0.1, 0.2, 0.25, 0.3, 0.35 y 0.4 mg GA/mL). Results are shown as mg of gallic acid equivalents per mL of infusion (mg GAE/mL).

The total flavonoid content was determined by the colorimetric method reported by Gutiérrez-Grijalva et al. [26]. Briefly, 10 µL of the sample was placed in a 96-well microplate; then 250 µL of distilled water was added, followed by 10 µL of 10% AlCl_3_. Finally, 10 µL of 1 M C_2_H_3_KO_2_ were added and left to incubate for 30 min. Then, absorbance was measured at 415 nm using a 96-well microplate reader. The total flavonoid content was quantified using a quercetin standard curve (0–0.4 mg/mL). Results are shown as mg equivalents of quercetin per mL of infusion (mg QE/mL infusion).

### 2.8. Total Tannin Quantification

Tannins were evaluated following the report by Vazquez-Olivo et al. [27]. Briefly, tannins were quantified using a catechin standard curve and expressed as milligrams of catechin equivalents per mL of infusion (mg CE/mL infusion). Next, the non-tannin phenolic content was measured by precipitating tannins with polyvinyl polypyrrolidone (PVPP), a tannin-binding agent, and the Folin–Ciocalteu method was used to determine the content of non-tannin phenolics. Finally, the total tannins were calculated by subtracting the non-tannin phenolics from the total reducing capacity (as an indicator of the total phenolic content, using a catechin standard curve) using the following equation:

Total tannin content = Total reducing capacity − non-tannin phenolics

#### Condensed and Hydrolyzable Tannin Content

The condensed tannin content was measured following the report of Heil et al. [28]. Briefly, 100 µL of the sample was mixed with 1 mL of 4-dimethylaminocinnamaldehyde (DMCA) solution (0.1% DMCA in methanol/hydrochloric acid 9:1 *v*/*v*). Absorbance was measured at 640 nm after 5 min of incubation at room temperature. The condensed tannin content was calculated using a catechin standard curve and expressed as milligrams of catechin equivalents per mL of infusion (mg CE/mL infusion). The hydrolyzable tannin content was calculated by subtracting the condensed tannin from the total tannin content.

Hydrolyzable tannins = total tannin content − condensed tannin content

### 2.9. Pancreatic Lipase Inhibition

The inhibitory rate of pancreatic lipase activity was measured as reported by Worsztynowicz et al. [29] with modifications reported in Gutiérrez-Grijalva, Antunes-Ricardo, Acosta-Estrada, Gutiérrez-Uribe and Basilio Heredia [26]. Briefly, the substrate *p*-nitrophenyl palmitate (*p*NPP) was used; the released p-nitrophenol by lipase activity was monitored at 410 nm. Briefly, 20 µL of sample and 20 µL of porcine lipase enzyme at 1 mg/mL in sodium phosphate buffer (0.1 M, pH 6.9) were incubated at 37 °C for 10 min. After incubation, 1800 µL of 0.1 M sodium phosphate buffer with sodium cholate (1.15 mg/mL) and Arabic gum (0.55 mg/mL) and 20 µL of *p*NPP in 0.01 M isopropanol were added and left to incubate for 10 min at 37 °C. Commercial orlistat was used as a positive control for pancreatic lipase inhibition at 20 mg/mL in phosphate buffer.

The inhibitory activity of pancreatic lipase was calculated using the following equation:

Pancreatic lipase inhibition (%) = ((Abs blank − Abs sample)/(Abs blank)) × 100(1)

where Abs blank is the absorbance of the blank, and Abs sample is the absorbance of the sample.

### 2.10. Statistical Analysis

Data were analyzed by one-way analysis of variance (ANOVA) followed by Tukey’s HSD test using the statistical software Minitab 17 (Minitab, LLC., State College, PA, USA). Statistical differences at the level *p* ≤ 0.05 were considered significant.

## 3. Results

### 3.1. Effect of Simulated In Vitro Gastrointestinal Digestion on Mistletoe Infusions

#### 3.1.1. Phenolic Compound Determination by UPLC-MS

The phenolic acid content in mistletoe infusions before and after the in vitro digestion process is shown in Table 1. We found three hydroxycinnamic acid derivatives (ferulic, coumaric, and caffeic acid), one hydroxybenzoic acid derivative (gallic acid), and one quinic acid in all samples of mistletoe. The UPLC-MS analysis showed that ferulic acid is the most abundant in undigested samples, ranging from 55.15 μg/mL in whole plant infusions to 60.77 μg/mL in stem infusions, followed by quinic acid with a total content of 52.61 μg/mL in whole plant infusions to 60.24 μg/mL in stem infusions (*p* ≤ 0.05). Stem infusions showed the overall highest phenolic content in undigested samples (*p* ≤ 0.05).

**Figure 1:**
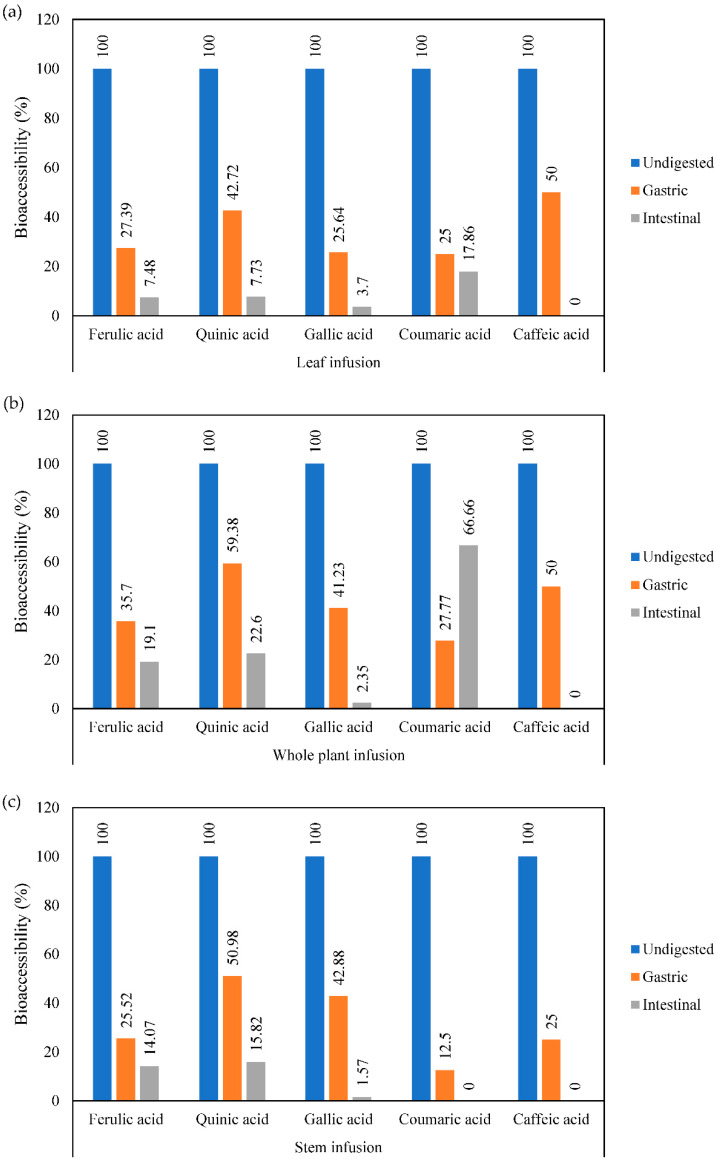
Bioaccessibility of phenolic acids from mistletoe (**a**) leaf infusion, (**b**) whole plant infusion, and (**c**) stem infusion subjected to simulated in vitro digestion.

Moreover, in Figure 1, we can appreciate the bioaccessibility values of the individual phenolics evaluated in undigested samples, gastric and intestinal digests. During the in vitro gastrointestinal digestion, our results showed that the most bioaccessible phenolics in the gastric phase in leaf infusions (Figure 1a) were caffeic and quinic acids, with 42.72% and 50.00%, respectively. On the other hand, the most bioaccessible phenolic at the intestinal phase was coumaric acid. Moreover, in whole plant infusions (Figure 1b), the most bioaccessible phenolic compounds in the gastric phase were quinic and caffeic acids, with values of 59.38% and 50.00%, respectively; while coumaric, ferulic, and quinic acids were the most bioaccessible at the end of the digestion process. Finally, in stem infusions (Figure 1c), quinic and gallic acids were the most bioaccessible in the gastric phase, with values of 50.98% and 42.88%, respectively. In addition, quinic and ferulic acids were the most stable at the end of the intestinal phase of digestion.

#### 3.1.2. Total Reducing Capacity and Total Flavonoid Content

**Figure 2:**
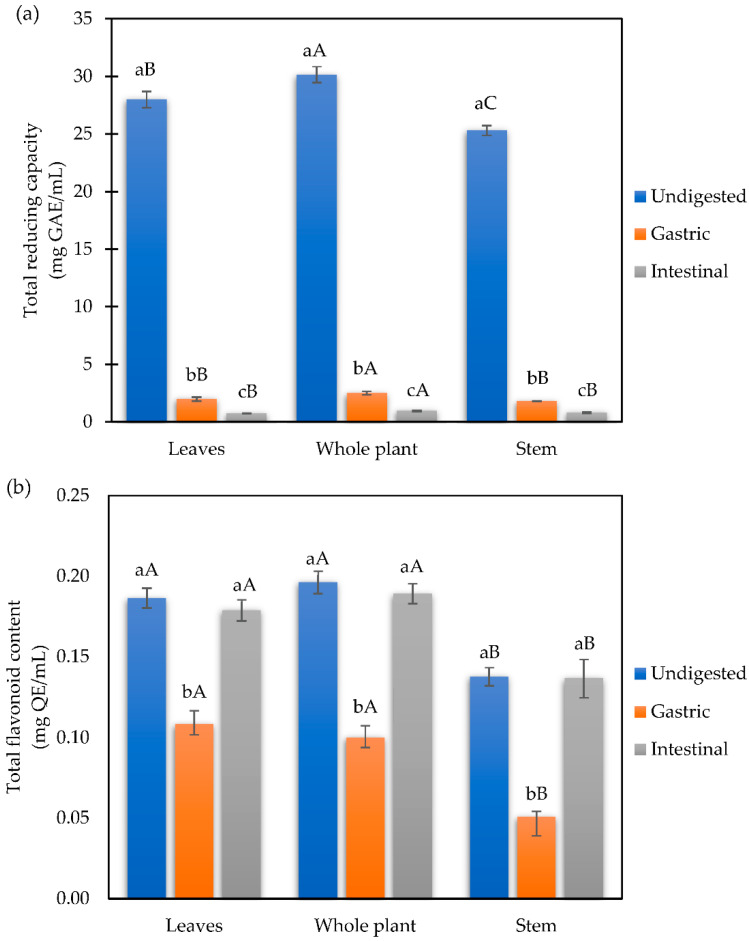
(**a**) Total reducing capacity and (**b**) total flavonoid content of mistletoe infusions subjected to simulated in vitro gastrointestinal digestion. Different letters indicate statistical significance (*p* < 0.01) by Tukey’s test. Data shown as means ± SD of three replicates (n = 3). Means that do not share capital letters show statistical differences between plant parts in each digestive phase by the Tukey test (*p* < 0.05).

The results of total reducing capacity (Figure 2a) showed that the highest proportion is found in the whole plant infusion (30.15 mg GAE/mL), followed by the leaf infusion (28 mg GAE/mL) and the stem infusion (25.30 mg GAE/mL). Moreover, the highest total flavonoid content (Figure 2b) was shown in the whole plant and leaf infusions with 0.20 and 0.19 mg QE/mL, respectively. During the in vitro gastrointestinal digestion, we observed decreased levels of total reducing capacity in all samples by the Folin–Ciocalteu method (Figure 2a), where more than 90% of the content was lost at the end of the intestinal phase. On the other hand, during the gastric phase of digestion, the total flavonoid content decreased significantly (Figure 2b); but at the end of the intestinal phase, the total flavonoid content increased significantly in infusions from all samples.

#### 3.1.3. Tannin Content

**Figure 3:**
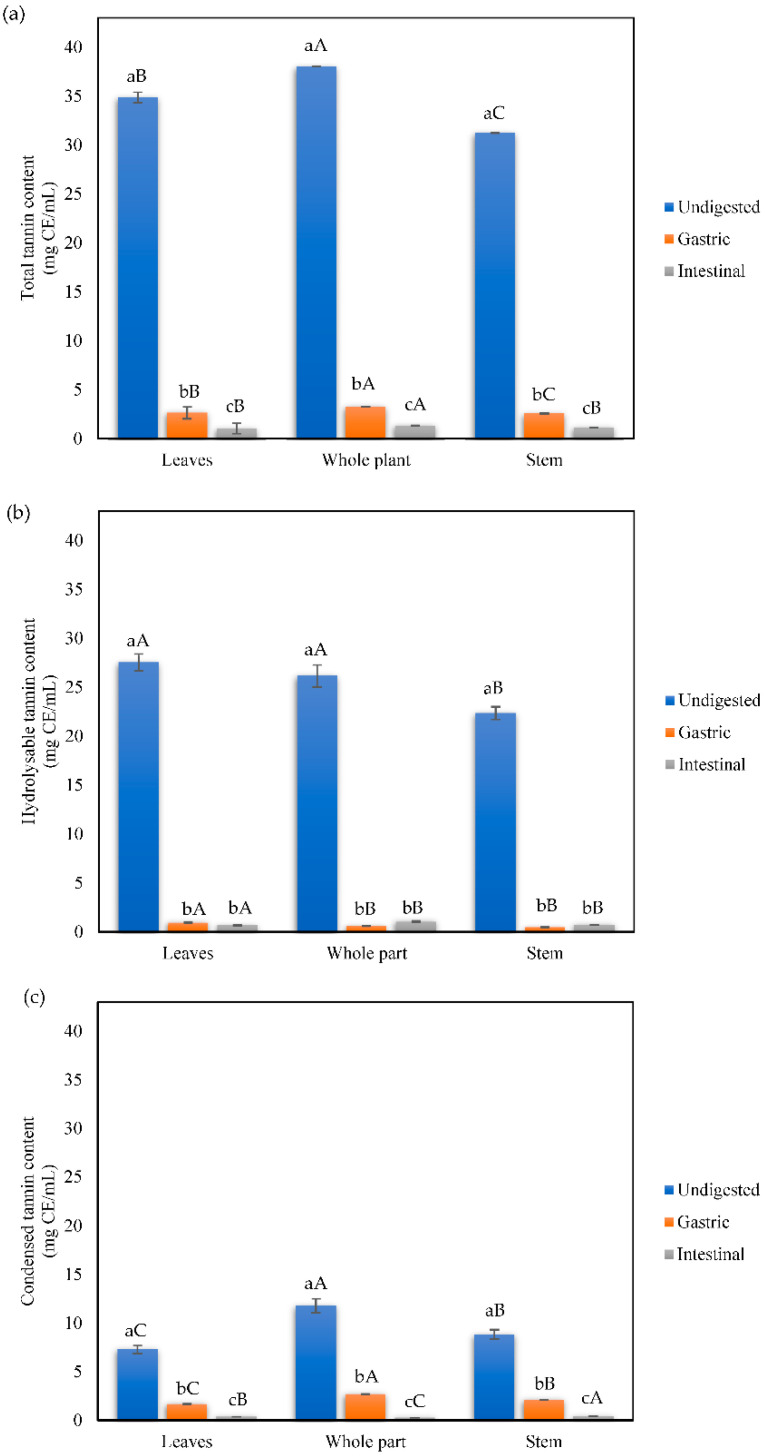
(**a**) Total, (**b**) hydrolyzable, and (**c**) condensed tannin content of mistletoe infusions subjected to simulated gastrointestinal digestion. Data shown as means ± SD values with at least three replicates (n = 3). Means that do not share lowercase letters are significantly different by the Tukey test (*p* < 0.05). Means that do not share capital letters show statistical differences between values per plant part in each digestive phase by the Tukey test (*p* < 0.05).

The total, condensed, and hydrolyzable tannin content is shown in Figure 3. In quantifying total tannins in the undigested infusions, the whole plant infusion showed the highest concentration with 38.00 mg CE/mL, followed by the leaf infusion with 34.86 mg CE/mL. Moreover, when we measured the hydrolyzable (Figure 3b) and condensed (Figure 3c) tannin content, the whole plant infusion showed the highest concentration. In general, total, condensed and hydrolyzable tannin content in infusions from all plant parts were negatively affected by the simulated digestion, with values ranging from 1.04 to 1.32 mg CE/mL, 0.27 to 0.4 mg CE/mL, and 0.66 to 0.72 mg CE/mL, respectively.

#### 3.1.4. Antioxidant Capacity

**Figure 4:**
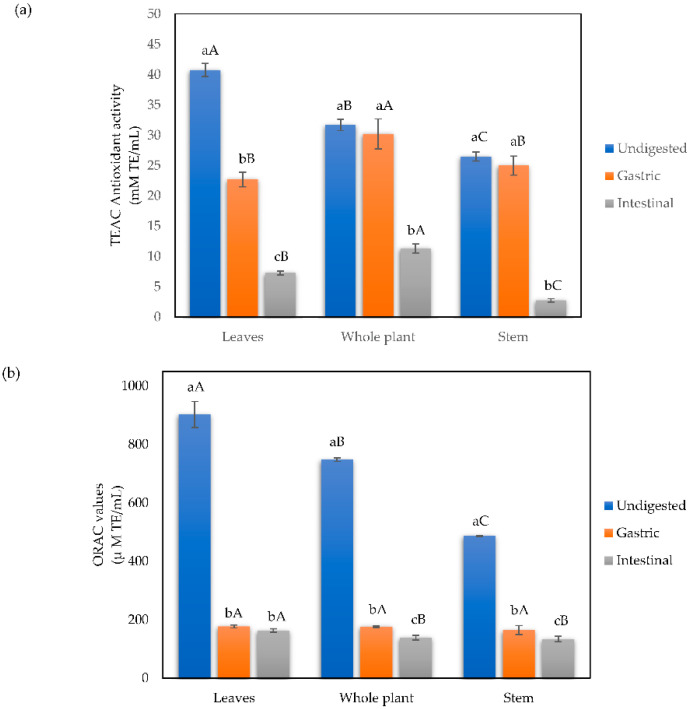
Antioxidant capacity by the (**a**) TEAC and (**b**) ORAC methods of leaves, whole plant, and stem mistletoe infusions subjected to simulated in vitro gastrointestinal digestion. Different letters indicate statistical significance (*p* < 0.01) by Tukey’s test. Data shown as means ± SD of three replicates (n = 3). Means that do not share capital letters show statistical differences between values per plant part in each digestive phase by the Tukey test (*p* < 0.05).

The results of the TEAC assay (Figure 4a) show that in the undigested infusions, the highest antioxidant capacity is found in the leaves (40.75 mmol TE/mL), followed by the whole plant infusion (31.69 mmol TE/mL) and finally by the stem (26.50 mmol TE/mL). Regarding the samples obtained from the digestive phases, the TEAC values of antioxidant capacity of the whole plant and stem infusions remained stable in the gastric phase, preserving 95.36% and 94.26% of its undigested values, respectively, concerning the undigested phase. On the other hand, the leaf infusion has a significant loss from the gastric phase (55.73%). In the recovered digested samples from the intestinal phase, the one that maintains a better radical inhibition is the infusions of the whole plant (35.75%), followed by the leaf infusion (17.86%), and finally, the stem infusion (10.41%).

The antioxidant capacity of undigested mistletoe infusions by the ORAC method (Figure 4b) follows the same pattern previously commented on in the TEAC method, with the leaf infusion having the highest antioxidant capacity (902.71 μmol TE/mL), followed by the whole plant infusion (747.72 μmol TE/mL) and the lowest capacity being found in the stem infusion (486.62 μmol TE/mL). In the samples recovered from the intestinal phase, there is a significant decrease in the antioxidant capacity measured by the ORAC method compared to the gastric phase, with the stem infusion being the one that best maintained its antioxidant capacity (33.88%), followed by the whole plant (23.62%) and finally the leaf infusion (19.64%). In the infusions recovered in the intestinal phase, the stem infusion is the one that better maintains its antioxidant capacity (27.53%) compared to the leaf and whole plant, which maintain similar capacities (18.10% and 18.66%, respectively).

#### 3.1.5. Inhibition of Pancreatic Lipase

**Figure 5:**
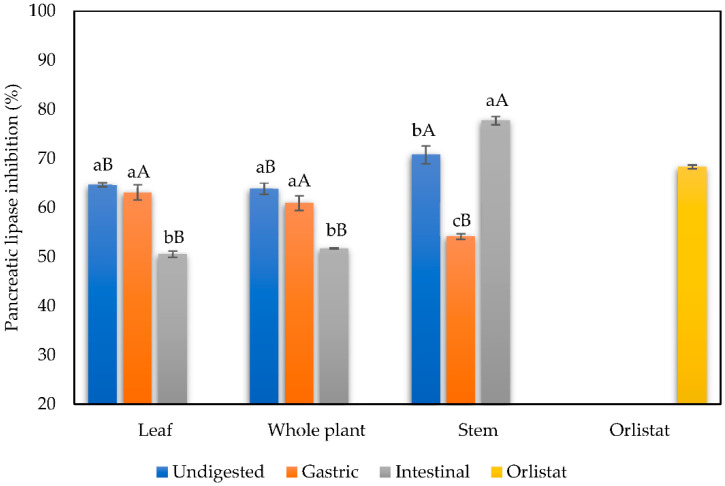
Pancreatic lipase inhibition of mistletoe infusions before and after gastrointestinal in vitro digestion. Data shown as means ± SD values with at least three replicates (n = 3). Means that do not share lowercase letters are significantly different by the Tukey test (*p* < 0.05). Means that do not share capital letters show statistical differences between values per plant part in each digestive phase by the Tukey test (*p* < 0.05).

The results of the inhibitory capacity of the pancreatic lipase enzyme expressed as a percentage are shown in Figure 5. The pancreatic lipase inhibitory capacity of undigested infusions was higher in stem infusions with 70.80% inhibition, followed by the whole plant and leaves infusions with 64.07% and 63.66%, respectively. After the simulated gastrointestinal in vitro digestion, at the end of the intestinal phase, the lipase inhibitory capacity decreased significantly in leaves and whole plant infusions; on the other hand, the stem infusions inhibitory activity of lipase increased at the end of this stage, increasing from 54.12% to 78.49% from the gastric to the intestinal phase.

## 4. Discussion

Different mistletoe species have been the subject of chemical characterization and ethnopharmacological studies; however, few reports have focused on evaluating the phenolic content of this plant species. In particular, the American mistletoe *P. brachystachyum* had not yet been explored, nor was the phytochemical content known. One of the few reports is the study published by Wang et al. [30], who identified six phenolic compounds in Peruvian *Phoradendron* sp., here the authors report that one of the most abundant phenolics is chlorogenic acids, which we did not find in our samples. This difference can be attributed to the geographical distribution of mistletoe and the species of the plant sample, as phenolic compounds can vary among species and can be affected by geographical distribution [31].

Moreover, a study by Furlan et al. [32] showed that the Brazilian mistletoe species *P. perrottetii* had a high content of quinic acid derivatives, which is consistent with our study, where quinic acid was the second most abundant metabolite in infusions from leaves, stem, and whole plant. In this sense, the authors mention that due to the hemiparasitic nature of mistletoe, it can modulate its phenolic constituents as the plant adapts to the host response, which usually defends itself by increasing its oxidative response by augmenting reactive oxygen species levels to cause local cell death and stop the parasitic invasion [32,33,34]. Thus, the host species and response can also affect the phytochemical profile of mistletoe. In our study, the high content of ferulic and quinic acids might be associated with the antioxidant *P. brachystachyum* response to the oxidative burst defense mechanism provoked by its host, *Prosopis juliflora* [32]. Furthermore, a study by García-García et al. [35] on *Phoradendron bollanum* showed the vast diversity of quinic acid esters in caffeoylquinic and feruloylquinic acids; however, the authors did not quantify the metabolites.

Several bioaccessibility reports have shown that one of the main factors affecting phenolic compounds’ stability during in vitro gastrointestinal digestion is the pH changes during each digestive stage and the possible interaction between the digestive enzymes and the metabolites in the sample [16,17]. This study showed that the phenolic content of mistletoe infusions is unstable when subjected to simulated gastrointestinal conditions. In addition, it has been reported that during simulated gastrointestinal digestion, the pH changes can cause deprotonation, degradation, and partial hydrolysis of phenolics [36,37,38,39]. Additionally, it is important to mention that bioaccessibility studies of mistletoe species (European or American) are scarce and up to date this is the first report that assesses this issue, thus, making comparison difficult.

On the other hand, in this study, we observed a decrease in the total reducing capacity in infusions from all samples during the simulated gastrointestinal digestion; this effect was not observed when we measured the total flavonoid content by the aluminum chloride method, as the total flavonoid concentration increased at the end of the intestinal phase. As previously mentioned, some polymerized phenolic compounds can partially hydrolyze during gastrointestinal digestion.

Moreover, it is important to mention that tannins are among the phytochemicals commonly found in mistletoe species and have been reported as inhibitors of digestive enzymes due to their high affinity to interacting with proteins [35,40,41,42]. Here, it is important to highlight that we found high content of total and hydrolyzable tannin content; so we hypothesize that some products of the partial hydrolysis of tannins are associated with the increased flavonoid content at the end of the intestinal stage of digestion [43].

Furthermore, the antioxidant capacity of mistletoe infusions from leaves, stems, and whole plants decreased after the simulated gastrointestinal digestion. This effect has been associated with the -OH loss caused by deprotonation triggered by pH changes in the gastrointestinal environment; this could reduce the interaction between the antioxidant from the sample and the target molecule during each antioxidant assay [39,44].

In the case of digests from the intestinal phase, leaves, and whole plant, they show significant decreases (49.65% and 51.08%, respectively) with respect to the gastric phase. However, the stem infusion in the intestinal phase significantly increased the percentage of inhibition of the lipase enzyme (78.49%) compared to its previous phase and the undigested infusion. The increase in lipase inhibition in stem infusions at the end of the digestion could be related to the increased total flavonoid content shown for this sample. In this case, it has been reported that the colorimetric flavonoid assay based on aluminum complex formation used here is more specific for flavonols and flavones, which could be formed during gastrointestinal digestion due to pH changes [45]. Moreover, the orlistat tablet (control drug) exerted an inhibition of 68.28%. Several factors could influence the increase in the inhibition of the enzyme pancreatic lipase by stem infusion in the intestinal phase; among them, it has been reported that the binding and inhibition of enzymes by antioxidants is more akin to larger polyphenolic compounds such as gallolysed and complex compounds such as tannins, as they can bind to larger binding sites, giving rise to allosteric denaturation, instead of binding to a single site of the protein or enzyme. In this, the composition of proline-rich protein groups is also a factor that facilitates enzymatic interaction and inhibition since some authors point out that the binding of antioxidants to prolines can occur through hydrogen bonds between the carbonyls of proline and phenolic hydroxyl groups. Likewise, in conditions with higher pH, the activity of these compounds increases, and therefore enzyme inhibition augments in the duodenal phase may compare to the gastric phase [42,46,47]. In this sense, it is noteworthy that the effectiveness of polyphenols is not necessarily related to their antioxidant potential [47], which is evident in this study because, in the specific case of stem infusion, the antioxidant capacity does not increase in intestinal phases. However, it does increase the biological activity for lipase inhibition. Moreover, to fully understand the biotransformation and metabolic fate of phenolics during gastrointestinal digestion, we suggest further untargeted metabolomic studies are performed, as this is an area of opportunity that has been explored scarcely.

## 5. Conclusions

Phenolic characterization in mistletoe species is scarce, and studies have mainly focused on the antiproliferative properties of terpenes such as moronic acid from this plant species. In this study, we found that the most abundant phenolic acids found in the leaf, stem, and whole plant infusions were ferulic and quinic acid. These metabolites have been reported as potent antioxidants, anti-inflammatories, and potential agents against hypercholesterolemia and hyperglycemia. However, these compounds are degraded during simulated gastrointestinal digestion mainly due to the pH conditions in each digestive phase, which could hinder their biological activity. Nonetheless, we found that mistletoe infusions still exert pancreatic lipase inhibitory activity after being subjected to an in vitro digestion process. In the case of stem infusions, the lipase inhibitory activity increased from 54.12% to 78.49% at the end of digestion. This could indicate the formation of new derivates during the digestive process that could increase the interaction with pancreatic lipase; this aspect is not yet fully evaluated in experimental research. Furthermore, more research is needed to evaluate the flavonoid constituents in mistletoe infusions, identify the active molecules against pancreatic lipase and escalate to in vivo studies. In addition, this study shows that mistletoe can be used as a source of phenolics in the development of functional foods with hypolipidemic potential.

## Figures and Tables

**Table 1:** Quantification and bioaccessibility of phenolic acids by UPLC-MS in mistletoe infusions before and after gastrointestinal in vitro digestion.

Part	Compound	Phenolic Content Before and After In Vitro Gastrointestinal Digestion (μg/mL)		
		Undigested	Gastric	Intestinal
Leaves	Ferulic acid	58.12 ± 1.40 ^a,AB^	15.92 ± 0.33 ^b,B^	4.35 ± 0.09 ^c,C^
	Quinic acid	54.98 ± 1.92 ^a,B^	23.49 ± 0.13 ^b,B^	4.25 ± 0.07 ^c,C^
	Gallic acid	9.71 ± 0.48 ^a,C^	2.49 ± 0.34 ^b,C^	0.36 ± 0.01 ^c,A^
	Coumaric acid	0.28 ± 0.00031 ^a,A^	0.07 ± 0.0015 ^b,A^	0.05 ± 0.0015 ^c,B^
	Caffeic acid	0.02 ± 0.0006 ^a,B^	0.01 ± 0.0006 ^b,A^	0.00 ± 0.00 ^c,A^
Whole plant	Ferulic acid	55.15 ± 1.17 ^a,B^	19.69 ± 1.61 ^b,A^	10.53 ± 0.40 ^c,A^
	Quinic acid	52.61 ± 0.49 ^a,B^	31.24 ± 1.03 ^b,A^	11.89 ± 0.30 ^c,A^
	Gallic acid	15.28 ± 0.88 ^a,B^	6.30 ± 0.41 ^b,B^	0.36 ± 0.01 ^c,A^
	Coumaric acid	0.18 ± 0.0020 ^a,B^	0.05 ± 0.0015 ^c,B^	0.12 ± 0.0010 ^b,A^
	Caffeic acid	0.02 ± 0.0006 ^a,B^	0.01 ± 0.0006 ^b,A^	0.00 ± 0.00 ^c,A^
Stem	Ferulic acid	60.77 ± 0.98 ^a,A^	15.51 ± 1.20 ^b,B^	8.55 ± 0.37 ^c,B^
	Quinic acid	60.24 ± 2.47 ^a,A^	30.71 ± 0.79 ^b,A^	9.53 ± 0.15 ^c,B^
	Gallic acid	22.90 ± 1.05 ^a,A^	9.82 ± 0.31 ^b,A^	0.36 ± 0.01 ^c,A^
	Coumaric acid	0.08 ± 0.0017 ^a,C^	0.01 ± 0.0006 ^b,C^	0.00 ± 0.00 ^c,C^
	Caffeic acid	0.04 ± 0.0006 ^a,A^	0.01 ± 0.0006 ^b,A^	0.00 ± 0.00 ^c,A^

Data shown as means ± SD values with at least three replicates (n = 3). Means that do not share lowercase letters are significantly different by the Tukey test (*p* < 0.05). Means that do not share capital letters show statistical differences between a phenolic acid per plant part in each digestive phase by the Tukey test (*p* < 0.05).

## Data Availability

Not applicable.
